# Evaluation of *HER2* Gene Amplification in Breast Cancer Using Nuclei Microarray *in Situ* Hybridization

**DOI:** 10.3390/ijms13055519

**Published:** 2012-05-08

**Authors:** Huiyong Jiang, Xiaoyan Bai, Cheng Zhang, Xuefeng Zhang

**Affiliations:** 1 Department of General Surgery, General Hospital of Shenyang Military Area Command, No. 83, Wenhua Road, Shenhe District, Shenyang 110840, China; E-Mails: huiyongj1@sohu.com (H.Y.J.); cz1971@126.com (C.Z.); 2 Division of Nephrology, Guangdong Provincial Institute of Nephrology, Nanfang Hospital, Southern Medical University, Guangzhou, Guangdong 510515, China; E-Mail: xiaoyanbai@fimmu.com

**Keywords:** breast cancer, microarray, nuclei microarray, fluorescence *in situ* hybridization (FISH), *HER2* gene

## Abstract

Fluorescence *in situ* hybridization (FISH) assay is considered the “gold standard” in evaluating *HER2/neu (HER2)* gene status. However, FISH detection is costly and time consuming. Thus, we established nuclei microarray with extracted intact nuclei from paraffin embedded breast cancer tissues for FISH detection. The nuclei microarray FISH (NMFISH) technology serves as a useful platform for analyzing *HER2* gene/chromosome 17 centromere ratio. We examined *HER2* gene status in 152 cases of invasive ductal carcinomas of the breast that were resected surgically with FISH and NMFISH. *HER2* gene amplification status was classified according to the guidelines of the American Society of Clinical Oncology and College of American Pathologists (ASCO/CAP). Comparison of the cut-off values for *HER2*/chromosome 17 centromere copy number ratio obtained by NMFISH and FISH showed that there was almost perfect agreement between the two methods (κ coefficient 0.920). The results of the two methods were almost consistent for the evaluation of *HER2* gene counts. The present study proved that NMFISH is comparable with FISH for evaluating *HER2* gene status. The use of nuclei microarray technology is highly efficient, time and reagent conserving and inexpensive.

## 1. Introduction

The *HER2* gene and its protein product, a 185-kDa receptor tyrosine kinase, were initially identified in a rat glioblastoma model [1]. The 185 kDa protein encodes P185 or erbB2 and plays an important role in the regulation of cell growth [2]. *HER2*/*neu* gene is the most frequently amplified gene in breast cancer (in 20~30% of cases) [3]. Its amplification and overexpression are associated with poor prognosis and resistance to cytotoxic drugs in breast cancer patients [4–6]. *HER2* gene amplification has been used for predicting prognosis and guiding treatment of invasive ductal carcinoma of the breast with trastuzumab [7–9]. Accurate evaluation of *HER2* status is important in the management of patients with candidacy for the HER2-targeting therapy.

Internationally, the algorithm for HER2 testing is to perform either an immunohistochemistry (IHC) method to assess HER2 overexpression, in which patients with equivocal HER2 expression (2+) are further tested to assess gene amplification of HER2 using the fluorescence *in situ* hybridization (FISH) method, or directly assess the HER2 status by the FISH method [10–12].

Researchers also use chromogenic *in situ* hybridization (CISH) or dual-color CISH for simultaneous detection of the *HER2* gene and the centromere region of chromosome 17 [13,14]. The FISH assay is technically reproducible and is a preferred method for evaluating *HER2* gene status [15].

Tissue microarrays, also called tissue chips [16], are an ordered array of tens of thousands of tissue cores in a single paraffin block. This allows for thousands of genes to be monitored simultaneously for expression level and comparisons to be made between many different tissues on one glass slide. Christopher *et al*. [17] introduced methods to make cultured cells into microarrays. It has the same advantage as tissue microarray. We established the nuclei microarray using nuclei for the FISH, mRNA *in situ* hybridization and cytochemistry study [18].

Nuclei microarray technology was used in the FISH detection of the *HER2* gene. This method allows for the simultaneous detection of multiple specimens with high comparability and cost efficiency. This study has investigated the concordance and correlation between fluorescence *in situ* hybridization (FISH) using conventional tissue sections and nuclei microarray fluorescence *in situ* hybridization in breast cancer patients.

## 2. Results and Discussion

### 2.1. Results

**Figure 1:**
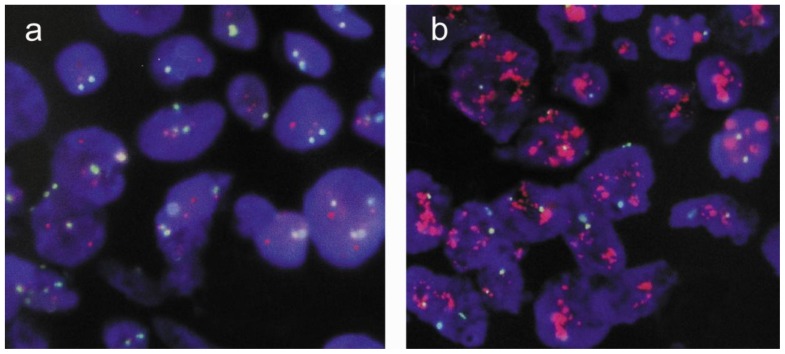
*HER2* gene status detected by NMFISH (HER2 signals red, CEP17 green) 100× objective. (**a**) *HER2* gene non-amplified; (**b**) *HER2* gene amplified. Nuclei Microarray FISH *versus* Conventional FISH

HER2 status for all samples was determined by conventional FISH and nuclei microarray FISH. Signals for both HER2 and chromosome 17 centromere were clearly detected using the nuclei microarray method (Figure 1). Separate signals could be counted without difficulty for non-amplified, equivocal, and amplified HER2 samples. Of the 152 cases analyzed, 42 were found to be amplified both by conventional FISH and Nuclei Microarray FISH (NMFISH) (ratio of *HER2*/CEP17 more than 2.2), giving an amplification rate of 27.6%.

Of the 152 cases, the NMFISH method detected HER2 amplification (HER2 ratio above 2.2) in 44 cases, equivocal (ratios from 1.8 to 2.2) in 22 cases and 86 cases showed non-amplified (ratios less than 1.8), the conventional FISH method detected HER2 amplification in 42 cases, equivocal in 23 cases and 87 cases showed non-amplified HER2. The agreement between NMFISH and conventional FISH was almost perfect: 95.4% (145/152), κ coefficient = 0.920 (Table 1).

There was no significant difference between the two methods using the McNemar–Bowker Test (*P =* 0.333).

There were two discrepant cases that showed non-amplification by nuclei microarray FISH but equivocal amplification by conventional FISH. Three cases showed equivocal by nuclei microarray FISH but non-amplified by conventional FISH and two showed amplified by nuclei microarray FISH but equivocal amplification by conventional FISH. The *HER2*/CEP17 ratios of these seven cases are detailed in Table 2. *HER2*/CEP17 ratios detected with the two diagnostic methods were very similar and close to the cut-off value. This may have caused the differences in diagnoses.

### 2.2. Discussion

Trastuzumab therapy has been used for the treatment of metastatic breast cancer for some years. Due to the development of adjuvant trastuzumab therapy, pathological laboratories are currently requested to perform HER2 status assays for all newly diagnosed breast cancers. The ASCO/CAP guidelines recommend the HER2 status to be optionally determined by IHC or FISH. In most laboratories, IHC is done first. In cases where the primary immunohistochemical result is equivocal at the protein level, such as an IHC score of 2+, faint staining or other artifacts, an additional test is strongly recommended to clarify the HER2 status at the genomic level to determine amplification or nonamplification. Thus, the need for reliable diagnostic and cost-effective *HER2* gene tests is rapidly increasing.

Tissue microarrays, also called tissue chips, are a novel technology invented by Konenen *et al*. [19], based on cDNA microarrays in 1998. It is a high throughput and resource conserving technology where tens of thousands of typical minute cylindrical tissue samples or cells from hundreds of different tumors are transferred to a new paraffin block. Tissue microarrays can be used in the detection of DNA, RNA or protein in various clinical or basic research areas [20,21].

Christopher *et al*. [17] introduced methods to make cultured cells into microarrays. This inspired us to make nuclei microarray with nuclei extracted from the paraffin embedded tissues for the FISH detection. The present study proved this method was feasible for the detection of *HER2* gene amplification in breast cancer.

During the process of making the nuclei microarray, 30-μm thick paraffin sections were cut for the extraction of nuclei instead of using the 4-μm thick sections used during the conventional FISH detection. This ensured that the nuclei were intact without being sectioned and the genetic DNA material was retained.

In the majority of the cases, the FISH method was performed successfully on nuclei microarray and almost perfect agreement with the FDA-approved HER2 FISH pharmDx method was revealed. Similar reagents and handling in the pretreatment, denaturation, hybridization and stringent washing steps were used in the two methods. One hundred samples can be detected simultaneously using this method with the same amount of reagents previously needed for one entire specimen, greatly reducing the cost.

The nuclei microarray FISH technology is advantageous for its high efficiency and low background compared to the conventional FISH using paraffin embedded tissue sections. The entire tissue section was used for the extraction of nuclei from multiple areas of the tumor. Each microarray disk contains more than five hundred cells [18], which are sufficient for the FISH detection.

During the conventional FISH detection using paraffin embedded tissue sections, variations can occur due to loss of the genetic DNA material occurring when the tissue blocks were sectioned. In the present study, we did not find this discrepancy since the ratios between HER2 signal and CEP17 were used to evaluate the *HER2* gene amplification in breast cancer. The probability of the signal losses for HER2 and CEP17 due to sectioning is the same in this experiment. Thus, the nuclei microarray FISH and conventional FISH are both applicable for detecting *HER2* gene amplification in breast cancer.

The present study compared nuclei microarray FISH and conventional FISH in detecting *HER2* gene amplification in breast cancer. The McNemar–Bowker test revealed that there was no significant difference between the two methods (*P =* 0.333). κ test revealed there was almost perfect agreement between the two methods (κ coefficient = 0.920). There are seven cases with discrepant results while the ratios are close to the diagnostic cut-off value. This may be caused by the heterogeneity of the tissue specimens.

## 3. Experimental Section

A retrospective study was conducted over one and a half years to reveal 152 cases of invasive ductal carcinomas of the breast (aged 31 to 83 years, median 49 years) that had been evaluated for the *HER2* gene by conventional FISH on formalin-fixed paraffin embedded tissue sections and nuclei microarray FISH using nuclei of breast cancer cells.

All patients underwent a radical operation and the diagnosis was confirmed in the Department of Pathology, General Hospital of Shenyang Military Area Command. The clinical stage was stage I–IIIC. Tissue blocks were available from all patients. Samples from patients with a previous history of breast cancer, having received neoadjuvant therapy, late staged metastatic cancer or multiple primary cancers were excluded from this study.

A stained section of each tumor sample was prepared from blocks to confirm the diagnosis. Representative tumor areas for nuclei extraction were selected to construct the nuclei microarray. An adjacent 4-μm thick section was also obtained for conventional FISH analysis.

### 3.1. Nuclei Microarray Construction

Methods for constructing nuclei microarrays (NMAs) and extraction of nuclei from paraffin-embedded tissue blocks have been described elsewhere [18,22]. Briefly, a blank cell microarray paraffin block was made in a 10 × 10 matrix using a manual arrayer (Beecher Instruments, Sun Prairie, WI, USA, MTA-1). Thirty-micrometer-thick sections were cut from the paraffin block and placed on a glass slide as a mold for holding nuclei suspensions. For each specimen, four pieces of 30-μm thick paraffin sections were cut and the tissue sections were placed into a 1.5 mL microcentrifuge tube. Nuclei were extracted after deparaffination and enzyme digestion [18]. During the process of nuclei extraction, tissue debris was separated from the nuclei and removed. This ensured a clear background in fluorescence *in situ* hybridization (FISH) testing. Cell density was adjusted to 1 × 10^4^ cells/μL with phosphate buffered saline (PBS, pH 7.4). Pretreated nuclei were injected into each well of the paraffin mold using a sample injector. This step is crucial to achieve the precise injection of 0.3 μL cell suspension into each well. The slides with cells were heated at 65 °C for one hour and then dewaxed in xylene twice for 40 and 20 minutes, respectively. The cell microarray was ready after open-air drying.

### 3.2. Nuclei Microarrays Fluorescence *in Situ* Hybridization (NMFISH)

NMFISH analysis was evaluated using the PathVysion HER2 DNA Probe Kit (Abbott-Vysis, Downers Grove, IL, USA). The hybridization mixture included a centromere 17 specific Spectrum Green-labeled DNA probe and *HER2*/*neu* specific Spectrum Orange-labeled DNA probe.

The NMA slides were dried in a 65 °C oven for one hour, fixed with methanol-glacial acetic acid (3:1) for one hour. After air drying, slides were placed in citrate buffer (pH 6.0) and incubated for 10 minutes in a microwave oven, then transferred to freshly prepared 0.4% pepsin solution (0.16 g pepsin, 2850 U/mg solid, in 40 mL of 0.9% sodium chloride, pH 1.5) and dehydrated through a series of graded ethanol. Ten microliters of probe solution, as recommended by the manufacturer, was applied to the slide, protected with a coverslip, and sealed with a continuous bead of rubber cement. Slides were processed with a HYBrite instrument (Vysis, Downers Grove, IL, USA) programmed with a melt temperature of 82 °C for 10 minutes and hybridization temperature of 37 °C for 16 hours. After hybridization, the coverslip was removed and the slides were washed in stringent wash buffer first briefly at room temperature then in a 65 °C hot solution for 10 minutes. The slides were counterstained with diamidinophenyl-indole. The samples were analyzed under a 100× oil immersion objective using an Olympus BX-61 fluorescence microscope with appropriate filters.

### 3.3. Conventional Fluorescence *in Situ* Hybridization (FISH)

FISH was performed on 4-μm sections using the same probe as NM FISH. Tissue pretreatment was performed using the paraffin pretreatment kit I (Gene Tech Company 02J02-032) as per the manufacturers’ instructions. For each case, thirty invasive tumor cells were counted by two different scorers (XB and HJ).

### 3.4. Evaluation of *HER2* Gene Amplification

*HER2* gene amplification status was classified according to the following criteria (ASCO/CAP) [11]. FISH signals were assessed by two independent assessors examining 30 non-overlapping nuclei for each tissue. The number of red signals (HER2) and green signals (CEP17) for each cell was recorded. Calculation of the *HER2*/CEP17 ratio was performed.

The results were graded as *HER2*/CEP17 ratio: Negative *HER2* gene amplification was defined as a *HER2*/CEP17 ratio of less than 1.8. Equivocal *HER2* gene amplification was defined as a *HER2*/CEP17 ratio between 1.8 and 2.2. Positive *HER2* gene amplification was defined as a *HER2* CEP17 ratio of more than 2.2.

### 3.5. Statistics

Comparisons between FISH using conventional paraffin embedded tissue sections and nuclei microarray FISH were analyzed using McNemar–Bowker Test. Concordance data obtained from both the conventional FISH and nuclei microarray FISH were determined. The κ statistic method was used to measure the agreement of *HER2*/CEP17 ratio between the two assays. The κ statistic evaluates the level of agreement after adjustment for agreement expected to occur by chance alone with a κ coefficient >0.80 indicating near-perfect agreement, values of 0.61 to 0.80 substantial agreement, values of 0.41 to 0.60 moderate agreement, values of 0.21 to 0.40 fair agreement, values >0 to 0.20 slight agreement, and values of 0 no agreement or a random association [23]. SPSS (version 13.0; SPSS Inc.: Chicago, IL, USA, 2004) was used for all statistical analysis. A *P* value less than 0.05 was regarded as statistically significant.

## 4. Conclusions

Nuclei microarrays can be used in Fluorescence *in situ* Hybridization in assessing *HER2* gene amplification in breast cancer. The use of nuclei microarrays methodology is highly efficient, time and reagent conserving and low cost. The disadvantage is that it takes time to construct the nuclei microarray. However, the time needed for constructing the nuclei microarray is a small fraction of the overall time compared to the time needed to carry out an experiment from sectioning the 152 paraffin embedded blocks, performing the FISH experiment on the slides, and imaging, processing and analyzing the samples. Nuclei microarray FISH technology produces reproducible and identical results when compared to conventional FISH. It is applicable for use in research. In clinical settings, if there were a large number of specimens needed to be detected by FISH, nuclear array FISH could be considered.

## Figures and Tables

**Table 1:** Comparison of *HER2* gene amplification status by fluorescence *in situ* hybridization (FISH) *vs*. nuclei microarray *in situ* hybridization.

		Nuclei Microarray FISH		

FISH	Non-Amplified *	Equivocal §	Amplified ‡	Total
Non-amplified *	84	3	0	87
Equivocal §	2	19	2	23
Amplified ‡	0	0	42	42
Total	86	22	44	152

Overall agreement: 0.954, κ coefficient = 0.920;

* *HER2*/CEP17 ratio < 1.8;

§ *HER2*/CEP17 ratio ≥ 1.8, < 2.2;

‡ *HER2*/CEP17 ratio ≥ 2.2.

**Table 2:** Discrepant results of mean copy number ratios of *HER2* gene and chromosome 17 centromere by fluorescence *in situ* hybridization (FISH) and nuclei microarray *in situ* hybridization (NMFISH) in seven breast cancer samples.

Case #	NMFISH/FISH Status	NMFISH	FISH
1#	Non-amplified/equivocal	1.74	1.86
2#	Non-amplified/equivocal	1.67	1.85
3#	Equivocal/Non-amplified	1.87	1.71
4#	Equivocal/Non-amplified	1.91	1.65
5#	Equivocal/Non-amplified	1.86	1.74
6#	Amplified/equivocal	2.39	2.15
7#	Amplified/equivocal	2.45	2.13
